# Relationships among the Internal Health Locus of Control, Mental Health Problems, and Subjective Well-Being of Adults in South Korea

**DOI:** 10.3390/healthcare9111588

**Published:** 2021-11-19

**Authors:** Sunhwa Shin, Eunhye Lee

**Affiliations:** Nursing Department, College of Nursing, Sahmyook University, Seoul 01795, Korea; leeeh@syu.ac.kr

**Keywords:** COVID-19, internal-external control, mental health, personal satisfaction

## Abstract

The purpose of this study was to confirm the relationship between internal health locus of control, mental health problems, and subjective well-being in adults during the prolonged COVID-19 pandemic. In particular, the mediating effect of mental health problems on the relationship between internal health locus of control and subjective well-being was examined. A cross-sectional descriptive design was conducted via online survey. The participants were 600 adults over 20 years of age living in South Korea. The collected data were analyzed using hierarchical regression analysis and SPSS Process Macro (Model 4). As a result of the study, the internal health locus of control had a significant negative effect on mental health problems. In addition, in the process of the internal health locus of control affecting subjective well-being, the mediating effect of mental health problems was significantly shown. In the period of an infectious disease pandemic such as COVID-19, it is necessary to establish a strong internal health locus of control of individuals and to promote monitoring and treatment introduction for those with a low internal health locus of control. In addition, it was discussed that controlling mental health problems can improve subjective well-being, which is life satisfaction and happiness.

## 1. Introduction

As COVID-19 became prevalent in 2020, it adversely affected public health around the world, but it is not just a physical health issue; fear and a phobia of infection are affecting the society as a whole [1]. It was reported that, as of 6 August 2020, confirmed cases of coronavirus disease 2019 (COVID-19) worldwide included 4,973,568 cases in the United States followed by 2,862,761 in Brazil and 1,963,239 in India. The virus’s mortality rate was shown to be more than 10% in Mexico, England, Italy, France, Belgium, and the Netherlands. In the case of South Korea, 14,499 people have been infected, and the number of confirmed deaths was 302 people, with a mortality rate of 2.1% and a cure rate of 93.1% [2]. As such, the spread of an emerging infectious disease such as COVID-19 is provoking fear, depression, and anxiety among the general public [3,4], causing a profound effect on everyday life [5].

Subjective well-being means the overall satisfaction of life [6], and a variety of negative experiences due to the COVID-19 pandemic could affect life satisfaction [4,7]. Since it seems that the stress caused by COVID-19 has a negative effect on the quality of life [8], the longer the threat of COVID-19 persists, the more likely it is that the quality of life or subjective well-being of the public may decrease. Therefore, it is worthwhile to look at the degree of subjective well-being experienced by ordinary people at the present time while unpredictable COVID-19 persists. In addition, it is necessary to look at various factors to improve subjective well-being.

Mental health problems increase when an infectious disease such as COVID-19 spreads. In South Korea, the residents of the Daegu/Gyeongbuk area, which has a high number of confirmed COVID-19 cases, experienced psychological distress [9]. As the number of confirmed COVID-19 cases in the Seoul and Gyeonggi area increased since the mass infection stemming from an Itaewon Club around May, the residents of Seoul/Gyeonggi also experienced similar psychological distress. Specifically in the Daegu area, 65.3% of the residents reported experiencing anxiety and depression [9]. Studies from another country reported that the overall symptoms of depression or anxiety has been increased not by the COVID-19 itself, but the financial and social distress associated with COVID-19 [10,11]. Since such psychological distress affects mental health and causes depression and anxiety [12], it is necessary to prepare for “mentaldemics”, wherein mental trauma is prevalent even after the infectious disease has been overcome [9,13]. Therefore, it is necessary to look at the level of stress and people’s mental health condition in light of the COVID-19 pandemic.

Health locus of control refers to the belief that an individual’s health is controlled by external and internal factors, and it serves an important role in predicting health behavior adherence [14]. Such health locus of control is associated with socioeconomic factors [14,15,16], and it acts as a factor in improving health behavior [15]. Through prior studies, it is known that the internal health locus of control is a leading factor affecting health behaviors, such as well-being [17] and mental health [18]. Since people’s beliefs about controlling their health status affects health behavior, it can be inferred that there will be differences in subjective well-being depending on internal health control at the time of the prevailing COVID-19 pandemic. Among the concepts of health locus of control, the internal health locus of control is an individual belief that one’s health or illness results from one’s own doing, willpower, or sustained efforts [14,15]. In a previous study, internal health locus of control had a significant effect on well-being [17,19], while another study reported a significant effect of health locus of control on mental health symptoms (depression, anxiety, stress) when examining the correlation between health locus of control and mental health problems [18]. In addition, health locus of control was reported to have a direct effect on the health behavior [15], and fear of COVID-19 increased psychological pain; these mental health problems have reduced life satisfaction [20]. This study assumed that the individual’s internal health locus of control would influence mental health problems and subjective well-being in the COVID-19 pandemic situation. Accordingly, mental health problems were designated as mediators in the correlation between internal health locus of control and subjective well-being.

Therefore, this study examined the mental health status of adults experiencing a pandemic crisis with the surge of a large number of confirmed COVID-19 cases and determined how internal health locus of control and mental health problems affected people’s subjective well-being, which is an indicator of life satisfaction. The purpose of this study was to identify the mediating effects of mental health problems in the relationship between internal health locus of control and subjective well-being.

## 2. Materials and Methods

### 2.1. Research Design

This study is a cross-sectional descriptive design conducted in adults to determine the relationship between the internal health locus of control, mental health problems, and subjective well-being experienced during COVID-19.

### 2.2. Study Participants

The participants of this study were adults over 20 years of age living in Seoul/Gyeonggi and Daegu/Gyeongbuk, where there were many confirmed cases of COVID-19. In this study, we commissioned the Macromill Embrain Company of 1.3 million panelists. Participants were extracted according to population-proportional allocation in consideration of region, gender, and age. A random e-mail was sent to the panelists based on population proportions. Among the panel, 695 completed the survey, and 95 people were removed by treating a certain section as an insincere response when a single number was used, or the response time was very short. Finally, data from 600 respondents (313 in Seoul/Gyeonggi, 287 in Daegu/Gyeongbuk) were used for analysis.

### 2.3. Instruments

#### 2.3.1. Internal Health Locus of Control

The internal health locus of control used a scale adapted by Kim [21] to the Multidimensional Health Locus of Control (MHLC) scale developed by Wallstone et al. [18]. The Health Control Committee scale used 6 items of the internal health locus of control (IHLC), which emphasizes that the responsibility for health is within the self, and 6 items of the chance health locus of control (CHLC), which emphasizes that people believe they cannot control their health or disease because those are determined by fate or chance. The chance health locus of control was scored inversely and added to the internal health locus of control, and the higher the score, the higher the internal health locus of control. In a previous study by Kim [21], the internal consistency coefficient (Cronbach’s) was IHLC 0.83, and in this study, IHLC 0.75 was observed.

#### 2.3.2. Mental Health Problems

Mental health problems were measured using the Brief Symptoms Inventory-18 (BSI-18) validated in Korea by Park et al. [22]. BSI-18 is a tool that Park et al. [22] validated as a shortened version of the Symptom Checklist-90-Revised (SCL-90-R) measurement tool developed by Derogatis [23]. In this study, mental health problems were measured by depression (6 items), anxiety (6 items), and somatization (6 items) experienced during COVID-19. Each item was rated on a Likert-type 5-point scale ranging from “not at all” (0 points) to “there are many” (4 points), and the higher the total score, the worse the mental health problem. In a previous study by Park et al. [22], the Cronbach’s α value for the reliability of the measurement tool was 0.89, and in this study, it was 0.95.

#### 2.3.3. Subjective Well-Being

Subjective well-being was measured using a measurement tool that was validated by Kim et al. [6], supplementing life expectation by using the Satisfaction with Life Scale (SWLS) developed by Diener et al. [24]. The subjective well-being measurement tool consists of 10 items, with 5 items focusing on life satisfaction and 5 focusing on life expectation. Each item is rated on a Likert-type 7-point scale ranging from “strongly agree” (7 points) to “strongly disagree” (1 point), and the higher the total score, the higher the subjective well-being. In a previous study by Kim et al. [6], the inner consistency coefficient (Cronbach’s α) was 0.84 for life satisfaction and 0.93 for life expectation. In this study, subjective well-being was 0.95.

### 2.4. Data Collection and Ethical Consideration

Prior to the data collection of this study, ethical approval was obtained from an institutional review board in Sahmyook University in Seoul. The data collection method was conducted by the panel survey company online because it is difficult to conduct face-to-face surveys due to the COVID-19 infectious disease. Data collection was conducted in September 2020. Since this is an anonymous online survey that does not allow the participants to receive written consent, the participants are able to proceed with the questionnaire by presenting a “subject description” online and checking the “consent” box after the subject is familiar with it. The description of the subject of the study addressed the purpose and procedure of the study, matters related to the suspension of the questionnaire, withdrawal of consent, interrupting the questionnaire completion at any time of the subject’s choice, and that there is no disadvantage due to interruption or withdrawal of the research participation. The company provided participation incentive to panelists who completed all the surveys.

### 2.5. Data Analysis

The data collected in this study were analyzed using the Process Procedure Macro expansion program (version 2.16.3) in SPSS 25.0 (IBM Corporation, Armonk, NY, USA). General characteristics and frequency analysis of major research variables and descriptive statistics are presented. The difference of research variables according to general characteristics was followed by independent *t*-test, and ANOVA with Scheffé test was conducted for post-hoc analysis. The relationship between the research variables was analyzed by Pearson’s correlation. The structural relationship between the research variables was analyzed using Model 4 in Process Procedure for SPSS developed based on regression analysis. The bootstrapping method was used to extract 10,000 samples repeatedly, and a 95% confidence interval (CI) was calculated; if 95% CI did not include “0”, the mediating effect of mental health problems was statistically significant [25].

## 3. Results

### 3.1. Differences in Research Variables according to General Characteristics

The general characteristics of the participation are as follows (Table 1). By gender, there were 302 males (50.3%) and 298 females (49.7%). The average age was 44.3 years (±13.15), with a minimum age of 20 and a maximum age of 72. By age group, 133 persons (23.0%) aged 40–49 years, 133 persons (22.2%) aged 50–59 years, and 121 persons (20.2%) aged 30–39 years were found in that order. The monthly income level was the most in the number of participants under 3 million won (320 persons, 53.3%). Of all the participants, 440 people (73.3%) had an occupation, and 344 people (57.3%) had no religion. A total of 276 people (46.0%) thought that COVID-19 was dangerous, 207 people (34.5%) thought that it was very dangerous. When asked whether they had close contact with a confirmed or suspected COVID-19 case, only 27 (4.5%) responded that they had. As for the time to search for COVID-19 information, 360 people (60.0%) took less than one hour, and 196 people (32.7%) took more than one hour, confirming that most of them have looked for information related to COVID-19.

The differences in internal health locus of control, mental health problems, and subjective well-being according to general characteristics were analyzed (Table 1).

The internal health locus of control for COVID-19 includes gender (*t* = 2.15, *p* = 0.032), age (F = 14.49, *p* < 0.001), marital status (*t* = −4.99, *p* < 0.001), religion (*t* = −3.50, *p* < 0.001), health status (F = 3.66, *p* = 0.026), risk perception for COVID-19 (F = 6.16, *p* = 0.002), close contact experience with confirmed patients (*t* = 2.14, *p* = 0.033), and COVID-19 information search time (F = 4.26, *p* = 0.015) showed significant differences. That is, males had a higher internal health locus of control than females, and the age group in their 60s had a higher internal health locus of control than other age groups, and the married group had a higher internal health locus of control than the unmarried group. Also, the religious group had a higher internal health locus of control than the non-religious group, and the healthy group had a higher internal health locus of control than the unhealthy group. The group that considered COVID-19 extremely dangerous had a higher internal health locus of control than the group who considered it slightly dangerous. The group with no experience of COVID-19 close contact had a higher internal health locus of control than the group with experience, and the group who spent more than an hour searching for COVID-19 information had a higher internal health locus of control than the group who did not search at all.

Mental health problems, including health status (F = 39.69, *p* < 0.001), close contact experience with confirmed patients (*t* = −2.55, *p* = 0.017), and COVID-19 information search time (F = 7.54, *p* = 0.001), showed significant differences. That is, the unhealthy group had higher mental health problems than the normal and healthy groups, and the group with close contact with COVID-19 had a higher mental health problem than the no experience group. Also, the group with more than 1 hour of searching for information about COVID-19 had higher mental health problems than the group with less than 1 hour and the group who did not search at all.

Subjective well-being, including education level (*t* = −2.87, *p* = 0.004), monthly income (*t* = −2.79, *p* = 0.005), occupation (*t* = −3.44, *p* < 0.001), religion (*t* = −5.26, *p* < 0.001), health status (F = 51.64, *p* < 0.001), and COVID-19 information search time (F = 3.53, *p* = 0.030), showed a significant difference. That is, the group with a university degree had higher subjective well-being than the group who graduated from high school, and the group with monthly income of 3 million won or more had higher subjective well-being than the group with 3 million won or less. In addition, the group with an occupation had higher subjective well-being than the group without an occupation, and the group with religion had higher subjective well-being than the group with no religion. Subjective well-being was higher in the healthy group than in the average and unhealthy groups.

### 3.2. Descriptive Statistics and Correlation of Research Variables

The internal health locus of control was 41.72 points (±5.77) on average, 15.30 points (±14.79) for mental health problems, and 40.19 points (±11.70) for subjective well-being. As a result of examining the skewness and kurtosis values of the study variables, all were distributed within ±2, satisfying the assumption of normal distribution (Table 2).

As for the correlation between the study variables, the internal health locus of control had a negative correlation with mental health problems (r = −0.27, *p* < 0.001), and a positive correlation with subjective well-being (r = 0.12, *p* < 0.003). Mental health problems had a negative correlation with subjective well-being (r = −0.26, *p* < 0.001) (Table 2).

### 3.3. Significance of the Mediation Effect of Mental Health Problems

**Figure 1:**
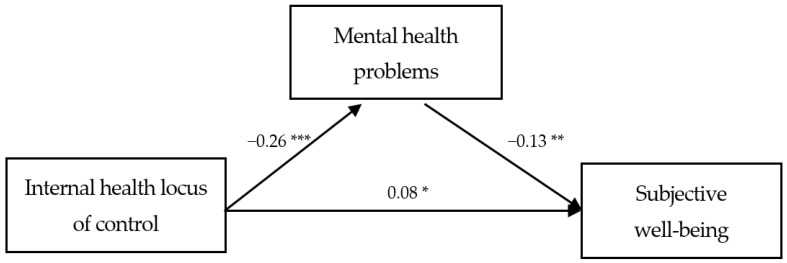
Study model. * *p* < 0.05; ** *p* < 0.01; *** *p* < 0.001.

In the process wherein the participants’ internal health locus of control affected their subjective well-being during COVID-19, the mediation effect of mental health problems was analyzed, and the results are presented in Table 3. Internal health locus of control displayed a significant direct effect on subjective well-being (**β** = 0.08, *p* = 0.044) after adjusting for potential covariates. Furthermore, the findings observed a significantly negative effect of internal health locus of control on mental health problems (**β** = −0.26, *p* < 0.001), and a significantly negative effect of mental health problems on subjective well-being (**β** = −0.13, *p* = 0.003). Thus, mental health problems showed a partially mediated effect on the relationship between internal health locus of control and subjective well-being. In the relation between internal health locus of control and subjective well-being, 95% confidence interval was identified for the significance of the mediating effect of mental health problems using bootstrapping. For the mediation effect of the internal health locus of control on the subjective well-being through mental health problems, the pathway coefficient was statistically significant (B = 0.08, 95% boot CI [0.02, 0.14]). The results of the study models are presented in Figure 1.

## 4. Discussion

This study intended to identify the relationships among internal health locus of control, mental health problems, and subjective well-being for adults in South Korea. In addition, it confirmed the mediation effect of mental health problems in the process of the internal health locus of control affecting subjective well-being.

In the process of the internal health locus of control affecting subjective well-being among general adults under the COVID-19 situation, mental health problems acted as a mediator. Although the internal health locus of control had a positive correlation with subjective well-being, its direct effects turned insignificant when mental health problems were introduced. Through this, it was confirmed that the internal health locus of control negatively affected mental health problems and that mental health problems were a mediating pathway that negatively affected subjective well-being. Although a previous study reported a direct effect of internal health locus of control on subjective well-being [17,19], this study verified the partial mediation effect of the internal health locus of control on subjective well-being by introducing mental health problems. Such an approach is thought to be meaningful. Considering the results of a previous study showing that those who believed in controlling their emotions and thoughts in a stressful situation were less likely to think negatively and cope well with the situation [26], it can be assumed that the internal health locus of control helps manage mental health. Although there are various factors that affect mental health problems, it was thought that the internal health locus of control can also act to reduce mental health problems. It is worth noting that the subjective well-being of those who have severe psychological distress due to the epidemic can be affected by mental health problems rather than directly by the internal health locus of control. Therefore, active management that includes professional psychological counseling or crisis intervention should be carried out for vulnerable classes experiencing mental health problems due to the COVID-19 pandemic.

Mental health problems were high in adults who had close contact with confirmed patients and those with more than one hour of information-searching time. Previous studies have reported that young or old age, female gender, marital status, education, economic level, and unemployment or loss of income were related to mental health problems [27,28,29]. During the COVID-19 pandemic period, for adults in the United States, women showed higher levels of mental health problems than men, and subjective assessments of fear, worry, and threat were related to mental health problems [12]. Stressors affecting mental health in the spread of infectious diseases included longer quarantine duration, fear of infection, frustration, boredom, inappropriate information, and stigma [30]. The pandemic situation of infectious diseases can cause mental health problems in vulnerable subjects, which are combined with demographic and psychological factors [31]. Therefore, it is essential to identify vulnerable subjects and apply timely therapeutic interventions. In addition, it is necessary to pay attention to not being exposed to the risk of mental health problems by providing accurate health information. It is required to establish a mental health system in preparation for emergencies of infectious diseases that threaten public health in the future.

In this study, men had a higher internal health locus of control than women, and adults aged 60 years and older had a higher internal health locus of control than young adults. In previous studies, factors such as age, education level, and monthly income were suggested as factors affecting the health locus of control [32,33]. In particular, it was found that people with a high internal health locus of control actively use health information and participate in education to perform well in health behaviors to solve problems [34]. In a pandemic situation that is prevalent around the world, the internal health locus of control can help a person maintain a good mentality and think rationally about the environment [35]. Several studies have reported a close relationship between internal health locus of control and mental health [18,35,36]. In a previous study on emerging adult migrants in China, the locus of control buffered the negative effects of perceived stress and strengthened the positive effect of social integration on psychological well-being [37]. Just as the internal locus of control acted as a factor influencing well-being in the moment of crisis of adult migrants [37], internal locus of control had a positive effect on subjective well-being of adults in the situation of the COVID-19 situation. People with a high internal health locus of control experienced less anxiety because they had a high level of independence in thinking and autonomy in self-determination [35], they tended to take responsibility for the consequences and events of their lives, and they made more of an effort and invested in themselves [18]. In this study, the increased searching related to COVID-19 and accordance with the sensitive risk perception of close contact means practicing desirable health behaviors for infectious diseases, which means that the internal health control locus is high. Therefore, the belief in developing the ability to control situations occurring in one’s life and taking responsibility for one’s own health can be seen as key factors in promoting health behavior.

In this study, the subjective well-being was lower in the case of a monthly income of 3 million won or less, no religion, and unhealthy status. In the UK, adults aged 30 to 59 and over 60 reported higher levels of well-being than young adults aged 18 to 29 under the COVID-19 situation, and women had lower well-being than men [38]. In China, about 74% of the general public reported a decrease in overall emotional well-being due to the coronavirus outbreak [31]. Medical workers who believe in conspiracy theories related to COVID-19 have increased anxiety and psychological distress, and decreased well-being, which is life satisfaction [39]. In the previous study, the importance of social experiences that reinforce positive emotions was emphasized by verifying the mediating effect of life satisfaction (well-being) in the relationship in which the public’s basic hopes affect anxiety [40]. As the COVID-19 pandemic has a longer-lasting impact on mental health than physical health, it is necessary to explore factors that threaten the well-being of community members in their daily lives [41]. Since subjective well-being is related to oneself [40], it is necessary to increase an individual’s internal empowerment to maintain a positive psychological state toward personal achievement and development. Also, in this study, the sub-factors of subjective well-being are composed of life satisfaction and life expectations. According to previous studies, it might be expected that higher life satisfaction may provide a psychological buffer against dangerous disorganization [42]. So, policies to promote mental health are needed to prevent adverse effects on subjective well-being, which is people’s life satisfaction.

As people today are experiencing fear and phobia due to the COVID-19 pandemic, strengthening the internal health locus of control will help people improve subjective well-being by practicing self-care, which maintains mental health and prevents the virus’s spread. Therefore, when it comes to promoting health education to the general public, a firm belief of self-health control should be included to improve mental health. In a recent study targeting adults in Spain, 20.8% reported severe anxiety and 27.5% had severe depression, while 66–80% reported that they practiced the recommended self-care guidelines [42]. At this point in time, when fear is increasing due to the spread of COVID-19, following the nationally recommended self-care guidelines is said to be essential. Therefore, it is necessary to provide correct information and practice continuous self-care in order to maintain the subjective well-being of the general public.

Since this study was conducted only in limited areas with a large number of COVID-19 confirmed cases, its results’ generalizability for the entire Korean population is limited. Thus, it is recommended to conduct a study comparing mental health problems, and subjective well-being by expanding the target areas to those with fewer confirmed cases or urban and rural areas. In addition, it is recommended to develop an effective educational program for the general public to improve mental health during the time of a pandemic crisis and to properly practice self-care. This study has a limitation in that it does not sufficiently reflect changes over time for major variables as it is a cross-sectional study. In addition, the possibility of interrelationships between major variables not considered in this study cannot be excluded. Therefore, in subsequent studies, it is necessary to establish and analyze various longitudinal research models in consideration of contextual interrelationships between major variables.

## 5. Conclusions

In this study, an online survey was conducted among adults in South Korea, where a large number of COVID-19 confirmed cases occurred. Through the analysis on the effect of internal health locus of control on subjective well-being among the adult participants, the pathway of the mediation effect of mental health problems was proven. Particularly in the COVID-19 situation, the internal health locus of control in adults helped reduce mental health problems. The internal health locus of control showed low scores in women, individual in their 20s, and unmarried people, and mental health problems were high in those in their 20s, unmarried, without religion, and single residents. Through these results, it was found that in the case of young adults in their 20s, with a large distribution of unmarried people, the internal health locus of control was low, and mental health problems were high. In this study, subjective well-being was low when monthly income was low, no religion was reported, and health conditions were unhealthy. These results suggest that the internal health focus of control, which can be regarded as an individual personality and cognitive strength, can play an important role in pandemic situations such as COVID-19. Therefore, it is necessary to increase internal health focus of control in daily life before a crisis occurs. Moreover, the study confirmed that controlling mental health problems can improve subjective well-being, which refers to people’s life satisfaction and happiness.

## Figures and Tables

**Table 1:** Difference in Measured Variables according to General Characteristics (*n* = 600).

Characteristics	Categories	*n* (%)	IHLC		MHP		SW	
			M ± SD	*t or* F (*p*)	M ± SD	*t or* F (*p*)	M ± SD	*t or* F (p)
Gender	Male	302 (50.3)	42.22 ± 6.27	2.15 (0.032)	14.61 ± 14.94	−1.15 (0.250)	40.73 ± 11.71	1.14 (0.255)
	Female	298 (49.7)	41.21 ± 5.16		16.00 ± 14.63		39.64 ± 11.68	
Age (yr)	20–29 (a)	107 (17.8)	39.50 ± 5.60	14.49 (<0.001) e > a, b, c, d e > d > a	16.61 ± 15.69	1.68 (0.154)	40.26 ± 11.40	1.52 (0.195)
	30–39 (b)	121 (20.2)	40.98 ± 5.70		15.91 ± 16.46		41.94 ± 11.85	
	40–49 (c)	138 (23.0)	41.04 ± 5.18		15.88 ± 14.87		40.64 ± 10.78	
	50–59 (d)	133 (22.2)	42.45 ± 4.76		15.68 ± 13.80		38.44 ± 11.90	
	≥60 (e)	101 (16.8)	44.93 ± 6.52		11.90 ± 12.42		39.71 ± 12.62	
Education level	High school	122 (20.3)	41.35 ± 5.97	−0.79 (0.429)	15.36 ± 14.56	0.05 (0.960)	37.49 ± 12.64	−2.87 (0.004)
	University	478 (79.7)	41.82 ± 5.72		15.28 ± 14.86		40.88 ± 11.36	
Marital status	Unmarried	210 (35.0)	40.15 ± 5.86	−4.99 (<0.001)	16.65 ± 15.81	1.65 (0.100)	39.90 ± 11.99	−0.45 (0.654)
	Married	390 (65.0)	42.57 ± 5.54		14.57 ± 14.17		40.35 ± 11.55	
Monthly income (10,000 won)	<300	320 (53.3)	41.50 ± 6.08	−0.99 (0.321)	15.47 ± 14.69	0.30 (0.761)	38.95 ± 12.22	−2.79 (0.005)
	≥300	280 (46.7)	41.97 ± 5.38		15.10 ± 14.93		41.61 ± 10.92	
Occupation	Don’t have	160 (26.7)	41.37 ± 5.53	−0.90 (0.366)	15.76 ± 14.26	0.46 (0.644)	37.41 ± 11.68	−3.55 (<0.001)
	Have	440 (73.3)	41.85 ± 5.85		15.13 ± 14.99		41.20 ± 11.55	
Religion	Don’t have	344 (57.3)	41.02 ± 5.64	−3.50 (<0.001)	15.66 ± 14.85	0.69 (0.490)	38.07 ± 11.70	−5.26 (<0.001)
	Have	256 (42.7)	42.67 ± 5.81		14.82 ± 14.72		43.04 ± 11.10	
Health status	Unhealthy (a)	78 (13.0)	41.91 ± 5.81	3.66 (0.026) c > b	26.73 ± 17.46	39.69 (<0.001) a > b > c	30.65 ± 11.71	51.64 (<0.001) c > b > a
	Average (b)	286 (47.7)	41.08 ± 5.76		16.00 ± 13.95		39.12 ± 10.83	
	Healthy (c)	236 (39.3)	42.44 ± 5.69		10.68 ± 12.49		44.64 ± 10.50	
Cohabitation	No (alone)	64 (10.7)	40.48 ± 6.22	−1.82 (0.069)	18.23 ± 15.15	1.68 (0.093)	38.03 ± 12.64	−1.56 (0.118)
	Yes	536 (89.3)	41.87 ± 5.70		14.95 ± 14.72		40.45 ± 11.57	
Risk perception for COVID-19	Extremely dangerous (a)	207 (34.5)	42.58 ± 5.99	6.16 (0.002) a > c	17.13 ± 15.98	2.47 (0.086)	40.11 ± 11.87	0.05 (0.953)
	Dangerous (b)	276 (46.0)	41.69 ± 5.33		14.21 ± 13.63		40.34 ± 11.47	
	Slightly dangerous (c)	117 (19.5)	40.26 ± 6.09		14.63 ± 15.03		39.97 ± 12.02	
COVID-19 close contact experience	No	573 (95.5)	41.83 ± 5.73	2.14 (0.033)	14.85 ± 14.37	−2.55 (0.017)	40.08 ± 11.66	−1.07 (0.283)
	Yes	27 (4.5)	39.41 ± 6.06		24.78 ± 19.99		42.56 ± 12.56	
COVID-19 information search time	Never (a)	44 (7.3)	40.05 ± 4.83	4.26 (0.015) c > a	10.20 ± 11.53	7.54 (0.001) c >a, b	37.93 ± 12.72	3.53 (0.030)
	Less than 1 h (b)	360 (60.0)	41.48 ± 5.72		14.31 ± 14.44		41.21 ± 11.59	
	More than 1 h (c)	196 (32.7)	42.55 ± 5.94		18.27 ± 15.55		38.83 ± 11.51	

IHLC = Internal health locus of control; MHP = mental health problems; SW = subjective well-being; M = mean; SD = standard deviation.

**Table 2:** Descriptive Statistics and Correlations of Measured Variables (*n* = 600).

Variables	IHLC	MHP	Min	Max	M ± SD	Skewness	Kurtosis
	r (*p*)						
IHLC			21.00	60.00	41.72 ± 5.77	0.32	0.37
MHP	−0.27 (<0.001)		0.00	71.00	15.30 ± 14.79	1.03	0.45
SW	0.12 (0.003)	−0.26 (<0.001)	10.00	70.00	40.19 ± 11.70	−0.22	0.01

IHLC = Internal health locus of control; MHP = mental health problems; SW = subjective well-being; M = mean; SD = standard deviation.

**Table 3:** Mediating Effects of Mental Health Problems in the Relationship between Internal Health Locus of Control and Subjective Well-Being (*n* = 600).

Path	B	SE	β	*t* (*p*)	Adj. R^2^	F (*p*)	Indirect Effect	
							Boot LLCI	Boot ULCI
IHLC → MHP	−0.47	0.07	−0.26	−6.76 (<0.001)	0.217	23.43 (<0.001)		
MHP → SW	−0.18	0.06	−0.13	−2.99 (0.003)	0.183	16.56 (<0.001)		
IHLC → SW	0.21	0.10	0.08	2.02 (0.044)				
IHLC → MHP → SW	0.08	0.03					0.02	0.14

IHLC = Internal health locus of control; MHP = mental health problems; SW = subjective well-being; B = unstandardized estimates; SE = standardized error; β = standardized estimates; Adj. R^2^ = adjusted R^2^; adjusted for gender, age, health status, and COVID-19 information search time; LLCI = lower level of the 95% confidence interval; ULCI = upper level of the 95% confidence interval.

## Data Availability

The data presented in this study are available on request to the authors.
